# Treatment outcomes of an integrated treatment model for patients with co-occurring opioid use and schizophrenia spectrum disorders

**DOI:** 10.3389/fpsyt.2026.1826744

**Published:** 2026-06-03

**Authors:** Yongjia Deng, Madison Seman, Charissa Ma, Seth Moomaw, James H. Berry, Kyle Durham-Vold, Kolbi Tonkovich, Jeremy Hustead, Sijin Wen, Wanhong Zheng

**Affiliations:** 1 West Virginia University School of Medicine, Morgantown, WV, United States; 2 University of Toronto, Toronto, ON, Canada; 3 Department of Behavioral Medicine and Psychiatry, West Virginia University School of Medicine, Morgantown, WV, United States; 4 Department of Epidemiology and Biostatistics, West Virginia University School of Public Health, Morgantown, WV, United States

**Keywords:** dual-diagnosis, opioid use disorder, oud, schizophrenia, schizophrenia spectrum, substance use disorder, thought disorder

## Abstract

**Background:**

Individuals with schizophrenia spectrum disorders are at elevated risk for developing opioid use disorder and often experience higher substance use rates and morbidity. However, integrated treatment models for such patients remain rare.

**Objective:**

This study addresses a gap in clinical knowledge by evaluating outcomes from a dual-diagnosis program tailored for patients with both disorders.

**Methods:**

This retrospective case series analyzed electronic medical record data from when patients were enrolled in the program through March 1^st^, 2025 or program discharge.

**Results:**

Seventeen patients attended at least one appointment. All patients had a diagnosis of opioid use disorder. Co-occurring psychotic disorders included schizoaffective disorder (13/17, 76%), schizophrenia (3/17, 18%), and unspecified psychotic disorder (1/17, 6%). On average, patients attended 58.8% of scheduled visits, and 47.1% remained actively enrolled as of 3/1/25. Medication adherence was high, with patients taking prescribed medications for opioid use disorder at 90.5% of visits and antipsychotics at 96.9% of visits. Compared to an equivalent pre-enrollment period, patients showed a significant reduction in emergency department visits (P=0.00051) and inpatient hospitalizations (P=0.00090).

**Conclusion:**

Findings suggest that the integrated treatment model could offer benefits for patients with co-occurring opioid use disorder/schizophrenia spectrum disorders; patients had significantly reduced emergency department visit frequency and frequency/duration of hospitalizations. Patients demonstrated high adherence to medications for opioid use disorder and antipsychotics; appointment attendance remains an area for improvement. Further research with larger samples and longer follow-up is warranted.

## Introduction

Individuals with opioid use disorder (OUD) are at elevated risk for developing schizophrenia spectrum disorders (1), often driven by self-medication, cognitive dysfunction, and social isolation. There is evidence of predisposition to this dual-diagnosis from gene variations in dopamine receptors implicated in the pathogenesis in both schizophrenia spectrum disorders and substance use disorders (SUD) (1). This dual-diagnosis presents challenges, as patients frequently experience worse outcomes, including higher rates of returning to substance use, poor engagement in care, and increased morbidity (1). A major barrier to effective treatment in this population is poor adherence to medications and outpatient follow-up (2). Low treatment compliance significantly impacts overall outcomes, often leading to repeated emergency department (ED) visits/hospital readmissions (3) and is further exacerbated by substance use.

Despite the challenges of this comorbidity, integrated treatment models that address both psychiatric disorders and SUDs remain rare in practice. This patient population is one that is often characterized by poor medication adherence and linkage to healthcare facilities (4). Accordingly, existing models of dual-diagnosis treatment generally involve the use of medications for opioid use disorder (MOUD), atypical antipsychotics, and psychosocial interventions in a coordinated care framework (1, 5, 6). For patients with thought disorders, some models suggest the avoidance of medications (such as those with anti-muscarinic properties) that can negatively affect cognition (7). However, the real-world application and long-term effectiveness of such models in patients with serious mental illness have been insufficiently studied.

The Comprehensive Opioid Addiction Treatment (COAT) model, developed in West Virginia (WV), combines MOUD, structured group therapy, and peer support in a phased outpatient setting. Research on outcomes have demonstrated the model’s success in achieving long-term retention, reducing opioid use, and adapting effectively to telepsychiatry during the Coronavirus disease 2019 pandemic (8). However, its impact on populations with psychotic disorders has not been well characterized.

This study seeks to address a gap in clinical knowledge and performance by evaluating outcomes from a COAT-based program tailored for individuals with co-occurring schizophrenia spectrum disorders and OUD. The TOAST program (Thought and Opioid Addiction Synergistic Treatment) is an integrated outpatient program designed to provide coordinated psychiatric and addiction care under one treatment team. Findings will inform clinicians on how integrated, recovery-oriented care can enhance engagement, reduce acute care utilization, and improve long-term outcomes in this high-risk population.

## Methods

This retrospective case series analyzed clinical data from patients enrolled in the TOAST program through March 1^st^, 2025, or until program discharge. Patients were referred into the program from inpatient and existing outpatient services at the institution. Community and self-referrals were also available. Patient were eligible for inclusion in the program if they were diagnosed with both OUD and a thought disorder. Patients were excluded from participation in the program if they were unable to commit to weekly in-person visits. Patients were enrolled at different time periods starting from March 2023. All data were obtained through manual review of electronic medical records (EMR) and securely stored through Research Electronic Data Capture (REDCap). Within the EMR data, specific data points were obtained as follows. Data on medication compilation was measured through a combination of patient self-reporting for oral medications and appointment compliance data for LAIs. Data on appointment compliance was measured through provider reports. Data on drug use was acquired through a combination of patient self-reporting and urine drug tests. Data on emergency department visits and hospitalizations (dates and durations) were also found through the patient’s medical history within their EMR.

The TOAST treatment team meets prior to group and discusses each patient at length, reviewing any admissions or other issues such as unreported substance use on random drug testing. Internal secure data sets are updated and reviewed weekly on patient symptoms, urine drug test results, medication changes, and group attendance.

The TOAST program is structured to deliver integrated thought disorder and addiction care in a group-based outpatient setting. Patients are seen weekly by an addiction psychiatrist in group sessions. Following psychiatric evaluation, they participate in group therapy focused on a mixture of psychoeducation and psychosocial rehabilitation, motivational interviewing, addiction relapse prevention, and coping skills training. Medications for opioid use disorder (MOUD), specifically buprenorphine-based products, are prescribed weekly. Antipsychotic medications are prescribed monthly, and long-acting injectables (LAIs) are administered on-site during scheduled visits when due. Random urine drug tests are conducted regularly, and patients are encouraged to self-report any substance use prior to testing.

Prior to each weekly appointment, a case manager will call/text/email patients to remind them of their appointments. A weekly TOAST meeting consists of a 30-minutepreclinical visit that includes urine drug testing and administration of any LAIs, typically antipsychotics or buprenorphine. The following 1-hour of group therapy is facilitated by a licensed therapist. The case manager and registered nurse also attend this session (they are not involved in therapy or prescribing). The final 30-minutes consists of the addiction psychiatrist interviewing all the patients in group to review their current medications, psychiatric symptoms, and recent substance use. In a normal week, this concludes the meeting; however, if a patient has additional needs or is in crisis, they can meet individually with any member of the treatment team.

Descriptive statistics based on the available medical records and program data sets were generated using REDCap’s native analytic tools. To assess changes associated with program participation, outcomes during enrollment were compared to a pre-enrollment period of equivalent duration. Paired t-tests were used to evaluate differences in variables including appointment attendance, hospitalizations, and medication adherence. A sample size of 17 patients provided 80% statistical power to detect a medium effect size (Cohen’s d = 0.725) using a two-sided paired t-test at a significance level of 0.05.

## Results

### Demographics

By March 1^st^, 2025, a total of 22 patients had been enrolled into the TOAST program. Of these, 17 attended at least one appointment, while the remaining five did not complete their intake visit and were subsequently lost to follow-up. Analyses were conducted on the 17 patients with available data; the five individuals who did not engage with the program were excluded due to insufficient information.

All included patients carried a diagnosis of opioid use disorder (OUD). Co-occurring SUDs were also prevalent, with tobacco use disorder present in 94% of patients, stimulant use disorder present in 71% of patients, and cannabis use disorder in 29%. Regarding psychotic disorders, 76% of patients were diagnosed with schizoaffective disorder (13/17), 18% with schizophrenia (3/17), and 6% with unspecified psychotic disorder (1/17).

See **Table 1** for additional demographic/diagnostic details including corresponding ICD-10 codes.

**Table 1:** Baseline patient characteristics (N = 17).

Participants	n=17*
**Age (mean, range)**	41.1 (29–56)
Gender	
Male	12 (70.59%)
Female	5 (29.41%)
Race	
White	16 (94.12%)
Native American	1 (5.88%)
Psychiatric Diagnosis:	
SUD	
Opioid (F11.20/F11.21)	17 (100%)
Stimulants (**F15.20)**	12 (70.59%)
Cannabis (F12.20)	5 (29.41%)
Hypnotic/Sedative (F13.20)	1 (5.88%)
Alcohol (F10.20)	6 (35.29%)
Tobacco (F17.200)	16 (94.12%)
Other Psychiatric Diagnoses	
Schizoaffective Disorder (F25.9)	13 (76.47%)
Schizophrenia (F20.9)	3 (17.65%)
Unspecified Psychotic Disorder (F29)	1 (5.88%)
MDD (F32.9/F33.9)	2 (11.76%)
PTSD (F43.10)	7 (41.18%)
ADHD (F90.9)	5 (29.41%)
GAD (F41.1)	6 (35.29%)
Panic Disorder (F41.0)	1 (5.88%)
Antipsychotic Prescription (n=16)	
Oral	12 (75.00%)
Long Acting Injectable	12 (75.00%)

*The cohort consisted of 22 patients, with data missing for five individuals.

### Retention and compliance

Patients enrolled in the TOAST program remained in treatment for an average of 315 ± 211.56 days. The majority demonstrated sustained engagement, with 88% participating for more than 90 days and 71% for over 180 days. On average, patients attended 55% ± 21% of their scheduled appointments, and 47.1% remained actively enrolled as of 3/1/25.

Among those who discontinued participation, the most common reason was loss to follow-up, often without a clearly documented cause. Despite challenges with appointment adherence, medication compliance was notably high. Patients were adherent to prescribed MOUD at 90.5% of visits and to antipsychotic medications at 96.9% of visits. Adherence was assessed during weekly encounters and documented in clinical progress notes.

All patients received MOUD, and all but one was prescribed antipsychotic medications during their time in the program. See **Table 2** for additional details on retention/compliance.

**Table 2:** Treatment compliance.

Treatment Compliance (n=17)*	Mean ± Standard Deviation	Median	Interquartile Range
Group Attendance			
Groups Scheduled	39.47 ± 26.28	33	16-54
Groups Attended	20.76 ± 15.46	19	11-26
Appointment Compliance^†^	0.55 ± 0.21	0.58	0.44-0.73
Medications for Opioid Use (MOUD) Compliance			
MOUD Non-Compliance Times	1.41 ± 2.50	0	0-2
MOUD Non-Compliance Calculation^‡^	0.095 ± 0.17	0	0-0.067
Antipsychotics Usage			
Non-Compliance Calculation^‖^	0.031 ± 0.080	0	0-0
Self-Reported Non-Compliance Times (n=15)	0.73 ± 1.83	0	0-0.5

*The cohort consisted of 22 patients, with data missing for five individuals.

†Appointment Compliance = Groups Attended/Scheduled.

‡MOUD Non-Compliance Calculation = Times Noted to be Non-compliant at Appointment/Total Group Sessions.

‖Oral (Verbal) Non-Compliance Calculation = Times Patient Self-reported Non-compliance/Total Group Sessions.

### Substance use patterns

Substance use data were collected for patients during their enrollment in the TOAST program, as well as for an equivalent pre-enrollment period when available. While enrolled, patients were seen weekly, allowing for more consistent monitoring. In contrast, most patients had less frequent contact with healthcare providers prior to enrollment, and drug use data from that period reflect only confirmed instances either through urine drug screening or patient self-report.

During program participation, patients experienced an average of 2.58 ± 4.17 recorded instances of opioid use annually, compared to 1.35 ± 2.17 recorded instances annually prior to enrollment. Only 29.4% of patients demonstrated a reduction in recorded opioid use frequency during the program compared to the pre-enrollment period.

Use of non-opioid substances - including alcohol, stimulants (including cocaine, methamphetamines, PCP, and MDMA), benzodiazepines, and marijuana - were notably higher. Four patients were recorded to have used benzodiazepines, four patients were recorded to have used alcohol, 12 were recorded to have used stimulants, and 11 were recorded to have used marijuana. Patient use of non-opioid substances was an average of 11.53 ± 11.50 times annually during enrollment, compared to 4.82 ± 4.97 times annually prior to enrollment. Only 5.9% of patients showed a reduction in non-opioid substance use rates during the program.

See **Table 3** for additional details on substance use patterns.

**Table 3:** Substance abstinence (days) and substance use summary.

Variable (n=17)*	Before TOAST		After TOAST	
	Mean ± SD	Median	Mean ± SD	Median
Opioid Use Instances	1.06 ± 1.78	0	2.35 ± 4.80	0
Longest Opioid Abstinence Times	**--**	--	251.71 ± 215.28	203
Non-Opioid Substance Use Instances	4.82 ± 4.97	4	11.53 ± 11.50	9
Longest Non-Opioid Abstinence Times	**--**	**--**	82.24 ± 132.57	35

*The cohort consisted of 22 patients, with data missing for five individuals.

### Emergency department visits and hospitalization patterns

Patients enrolled in the TOAST program experienced an average of 2.45 ± 5.11 ED visits per year during their time in the program, compared to 6.20 ± 8.77 visits annually during an equivalent pre-enrollment period. A two-tailed paired t-test revealed a statistically significant reduction in ED visits following program enrollment (P=0.00051).

Similarly, the average number of hospitalizations per year decreased from 3.52 ± 4.53 prior to enrollment to 1.51 ± 2.27 during participation in TOAST. Patients also spent fewer days hospitalized annually - 16.33 ± 30.99 days during enrollment versus 30.57 ± 37.05 days prior. Statistical analysis confirmed significant reductions in both hospitalization frequency (P=0.00090) following enrollment. There was a decreasing trend on the change in duration of hospitalization in TOAST using a two-tailed paired t-test (P=0.067) while it was significant with a one-tailed paired t-test (P=0.034).

**Figure 1:**
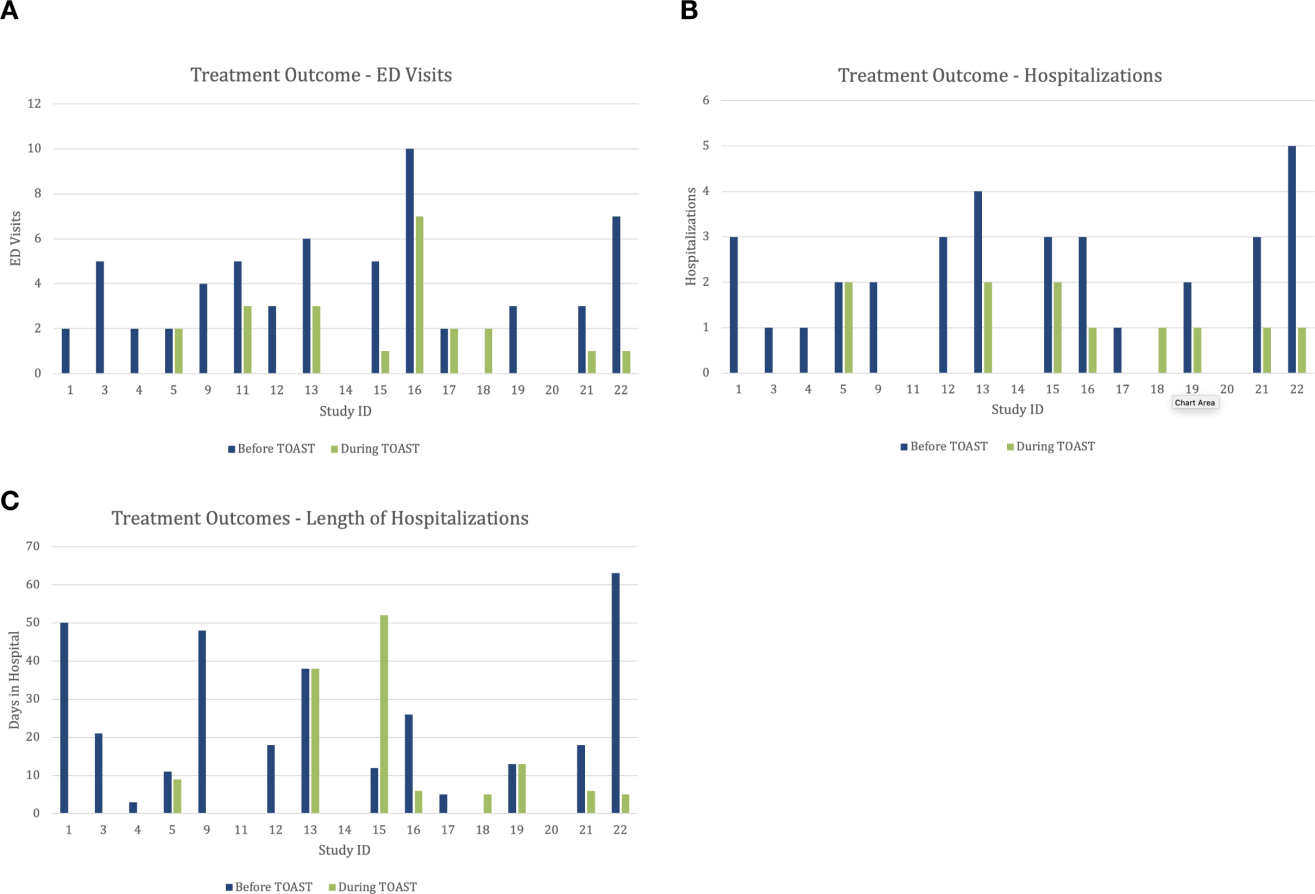
Data for ED visits **(A)**, hospitalizations **(B)**, and length of hospitalizations **(C)** for each of the 17 patients that successfully entered the program. The grey bar corresponds to the period in which a patient was actively participating in TOAST (up until 3/1/25) while the blue bar corresponds to an equivalent period of time prior to participating in TOAST. Each study ID represents an individual patient. Study IDs not represented on the horizontal axis (2, 6, 7, 8, 10) are patients who were referred to the program but never attended their intake appointment.

A graphical summary of these findings is presented in **Figure 1**.

## Discussion

### Retention and compliance

Medication and appointment adherence remain among the most significant challenges in the psychiatric care of individuals with schizophrenia. A meta-analysis of appointment attendance in this population reported missed visit rates ranging from 20% to 67% (9). In our study, the average appointment compliance rate of 55% aligns with this range. Although patients did not consistently provide reasons for missed appointments, prior research offers insight into contributing factors. Individuals with schizophrenia are disproportionately affected by poverty, with some studies indicating that over 80% live below the poverty line (10). Additionally, they tend to have smaller social networks (11), which may limit access to reliable transportation—whether through personal vehicles or support from family and friends.

The TOAST program currently does not offer telemedicine services, making transportation a prerequisite for participation. West Virginia University (WVU) Medicine partners with a company to provide transportation assistance; however, this service requires appointments to be scheduled at least five business days in advance, and punctuality is not guaranteed. These logistical barriers, combined with socioeconomic challenges, likely contribute to the observed appointment compliance rate. Future initiatives to address this could include offering a telemedicine option as well as providing more transportation options pending funding.

Retention in treatment is more complex for individuals with schizophrenia and OUD. Integrated programs addressing both conditions are rare, and data on long-term retention are limited. One study reported that 81% of patients with psychosis and co-occurring OUD remained in treatment with methadone after 12 months (12). In contrast, national data on buprenorphine-based outpatient treatment for OUD show that only about 20% of patients remain in care beyond 180 days (13). WVU’s COAT program reported a 48.4% retention rate beyond 90 days (14). Common reasons for early discontinuation include transfers to other providers, legal issues, and noncompliance with program requirements (15). For patients with schizophrenia, co-occurring substance use is the strongest predictor of early dropout (16).

The TOAST program demonstrated encouraging retention outcomes, with 88% of patients remaining in treatment beyond 90 days and 71% beyond 180 days—exceeding national averages. The program’s integrated model, which allows patients to receive care for both OUD and schizophrenia within a single setting, may reduce the likelihood of care fragmentation and improve continuity. By simultaneously addressing comorbid substance use, TOAST mitigates a key risk factor for disengagement. Despite these strengths, long-term retention remains a challenge; only 47.1% of participants were still enrolled at the time of data analysis, which is concerning given the program’s goal of lifelong treatment.

Medication adherence is another critical issue in this population. The Clinical Antipsychotic Trials of Intervention Effectiveness (CATIE) trial found that 74% of patients discontinued antipsychotic medications within 18 months of initiation (2). Among Medicaid beneficiaries with schizophrenia, only 41% were adherent to antipsychotic treatment (17). Adherence varies by formulation: one study reported 76% adherence among patients receiving LAIs, compared to 32% for oral medications (18). Common barriers to adherence include poor insight, substance use, side effects, and negative attitudes toward medication (19).

Adherence to MOUD is similarly problematic. A study involving over 20,000 patients found that only 40% met the threshold of taking MOUD as prescribed on at least 80% of days (20). Common reasons for buprenorphine noncompliance in literature include social situations (homelessness, lack of transport, *etc*), use of other illicit substances, diverting medications, and forgetfulness (21).

In contrast, patients in the TOAST program demonstrated high medication adherence, with compliance rates of 90.5% for MOUD and 96.9% for antipsychotics. Several program features likely contributed to these outcomes. By treating co-occurring substance use and schizophrenia concurrently, TOAST addresses a major barrier to antipsychotic adherence. The program also educates patients on and provides alternative formulations such as long acting injectables, which reduce the burden of daily medication management.

### Substance use patterns

During data analysis, an unexpected increase in non-opioid substance use was observed among patients enrolled in the TOAST program, while opioid use rates remained statistically unchanged. We believe this seemingly paradoxical finding is best explained by a combination of prior underreporting and surveillance bias.

Patients with SUDs often underreport their substance use, particularly in the absence of confirmatory mechanisms such as mandatory urine drug testing (22). This tendency is also well-documented among individuals with schizophrenia, especially those with greater neurocognitive impairments (23). In the TOAST program, random urine drug tests are routinely conducted, significantly increasing the likelihood of detecting undisclosed substance use. Patients are encouraged to self-report any recent alcohol or drug use prior to testing and is dealt with in a non-judgmental and therapeutic manner, reducing the incentive to conceal use. If substance use occurs, the patient does not face any potential for group expulsion; rather, a plan is formulated to reduce further use.

Additionally, the program’s structure - where patients are consistently seen by the same physician - helps foster therapeutic rapport and trust, which may further promote honest disclosure (24). Weekly in-person sessions also increase the frequency of provider-patient interactions compared to pre-enrollment care, enhancing the accuracy/completeness of substance use documentation. This increased monitoring likely contributes to surveillance bias, whereby more frequent contact leads to increased detection of substance use.

Importantly, TOAST’s group therapy sessions, which emphasize motivational interviewing and psychoeducation, may also play a key role in encouraging patients to be forthcoming about their substance use. Through group sessions, patients can learn from their peers who may have similar life experiences and gain an informal support network through the groups. There is evidence to show that group-based therapy sessions can provide advantages over individual therapy such as increased abstinence (25). These therapeutic approaches are designed to reduce stigma, build insight, and promote open communication, thereby creating a supportive environment that facilitates reporting of substance use.

Together, these factors - enhanced monitoring, therapeutic continuity, and a psychologically safe group setting - likely account for the observed increase in documented non-opioid substance use during program participation, rather than reflecting a true increase in usage behavior.

However, there is also the possibility that the increase in non-opioid substance use is genuine rather than an observational anomaly. While the research team does not believe this to be the case for our patients, it is not without precedent in the research. One study of adolescent patients in group therapy settings found that patients who interacted with other patients who glorified or encouraged substance use were more likely to engage in substance use later on (26). While our providers carefully control group discussions to keep the patients on topic, there is nothing stopping patients from meeting with each other outside of the clinical setting. If so inclined, patients could potentially share information with each other on preferred methods of administering substances as well as ways to acquire them. If patients are glorifying or encouraging substance use in that setting, it could potentially result in higher use despite efforts to avoid that.

Additionally, many large studies typically find that a small proportion of patients in substance use treatment end up using a greater amount of substances than prior to participation without any clear reason (27). Given the small sample size of this study, it is possible that patients inclined towards this may have been oversampled. Both social contagion and iatrogenic effects are possible explanations for the observed increase in non-opioid substance use.

### ED visits and hospitalization patterns

Patients who participated in TOAST were noted to have lower frequencies/durations of hospitalization and ED visits during time in the program compared to before. In general, patients with schizophrenia (3) and OUD (28) are known to utilize ED services at higher rates than the general public. One study found patients with schizophrenia to have 2.27 times higher odds of ED utilization compared to matched controls (29) and another study in the UK found that OUD patients had 2.80 times higher odds compared to the general public (30). Additionally, both groups are more likely to be hospitalized with users of illicit substances and schizophrenic patients having respectively 2.2 and 3.26 times higher odds of hospitalization compared to the general public (3, 31, 32). While treatment for each condition individually has been shown to reduce ED visits and hospitalizations (33, 34), there is evidence that dual-diagnosis programs can be effective as well with one pilot study finding a 74% reduction in hospitalization days in patients with comorbid substance use and schizophrenia (35). The reduction in hospitalization rates/days and ED visits experienced by patients upon participation in the TOAST program is in line with what has previously been documented in single-diagnosis treatment models as well as rarer dual-diagnosis treatment models.

### Limitations and future directions

This study has several limitations. First, the relatively small sample size of 17 patients limits the generalizability of the findings. The TOAST program is a relatively new initiative, launched in 2023, and the dual-diagnosis population it serves is not widely represented within the metropolitan area where the study was conducted. Additionally, there is a degree of selection bias within our results. Only patients who were deemed appropriate and capable of following with a provider in an outpatient setting were referred to the program which would likely exclude patients with more severe psychiatric symptoms. Additionally, the results of our study only reflect the 17 patients who were able to attend at least one appointment; there were an additional five patients who were referred but never attended any appointments for various reasons. Some cited transportation issues while others were lost to follow-up following referral. It is possible the factors that prevented these patients from attending their appointments may have affected their outcomes as well had we been able to follow-up with them.

Second, the quality and completeness of medical records prior to TOAST enrollment were limited. Many patients had infrequent contact with healthcare providers before entering the program, resulting in under-documentation of substance use and other clinical variables. Consequently, pre-enrollment data likely underestimates the true extent of substance use and healthcare utilization at the time.

Third, as a retrospective case series using patients as their own controls, the study is subject to confounding variables that cannot be fully accounted for. Factors such as changes in social support, housing stability, or access to other services may have influenced outcomes independently of program participation. It is known that environmental stressors can result in increased substance use and improvement of these factors could also conversely result in reduced substance use (36). Accordingly, factors unrelated to the TOAST program, including the natural course and history of chronic mental illnesses, may have had substantial influence on the outcomes of our participants.

Finally, due to the retrospective nature of the study, diagnoses assigned to participants represent pre-existing diagnoses given by a licensed psychiatrist through clinical judgement prior to research participation rather than research−grade, protocol−driven diagnostic reassessments. As part of this clinical judgement, the psychiatrist reviewed the patient’s medical history, psychiatric history, substance use history, history of laboratory testing, and neuroimaging prior to giving any diagnoses.

The combination of small sample size, potential selection bias, incompleteness of medical records, and the retrospective study design do potentially limit the generalizability of results and create the possibility of false positive results.

In the present study, we did not have detailed socioeconomic data available for each participant. Although we currently attempt to enhance follow−up compliance by contacting patients via phone and EMR-based messages, future work would benefit from more systematic collection of socioeconomic variables to better understand factors influencing follow−up participation. In addition, future research should aim to validate these preliminary findings through studies with larger sample sizes and prospective designs. Longitudinal follow-up of the current cohort may also provide valuable insights into long-term outcomes, treatment durability, and predictors of sustained engagement in integrated care models.

## Conclusion

This retrospective study evaluated the TOAST program, a novel outpatient model designed to treat individuals with co-occurring OUD and schizophrenia spectrum disorders. Integrated treatment approaches for this dual-diagnosis population are urgently needed yet remain underrepresented in the literature. This study contributes to a limited but growing body of research by providing early evidence of the program’s real-world impact on healthcare utilization and medication adherence.

Preliminary findings suggest that the TOAST integrated care model is associated with significant reductions in ED visits, as well as the frequency/duration of hospitalizations. Although appointment attendance remains an area for improvement, patient engagement was comparable to other outpatient programs targeting similar populations. Notably, medication adherence for MOUD and antipsychotics were high.

Further research is warranted to validate these findings through larger sample sizes and longer-term follow-up and to explore strategies to enhance patient engagement/retention. Overall, the TOAST program demonstrates the potential of integrated, recovery-oriented care to improve outcomes in complex psychiatric populations and underscores the need for continued innovation in dual-diagnosis treatment models.

## Data Availability

The datasets presented in this article are not readily available due to patient confidentiality. Requests to access these datasets should be directed to wzheng@hsc.wvu.edu.
